# 3D laparoscopic surgery: a prospective clinical trial

**DOI:** 10.18632/oncotarget.24669

**Published:** 2018-04-03

**Authors:** Antonino Agrusa, Giuseppe Di Buono, Salvatore Buscemi, Gaspare Cucinella, Giorgio Romano, Gaspare Gulotta

**Affiliations:** ^1^ Department of Surgical, Oncological and Stomatological Disciplines, University of Palermo, Palermo, Italy; ^2^ Department of Obstetrics and Gynecology, University of Palermo, Palermo, Italy

**Keywords:** 3D laparoscopy, 2D laparoscopy, laparoscopic surgery, laparoscopic abdominal surgery, three-dimensional vision

## Abstract

Since it’s introduction, laparoscopic surgery represented a real revolution in clinical practice. The use of a new generation three-dimensional (3D) HD laparoscopic system can be considered a favorable “hybrid” made by combining two different elements: feasibility and diffusion of laparoscopy and improved quality of vision. In this study we report our clinical experience with use of three-dimensional (3D) HD vision system for laparoscopic surgery. Between 2013 and 2017 a prospective cohort study was conducted at the University Hospital of Palermo. We considered 163 patients underwent to laparoscopic three-dimensional (3D) HD surgery for various indications. This 3D-group was compared to a retrospective-prospective control group of patients who underwent the same surgical procedures. Considerating specific surgical procedures there is no significant difference in term of age and gender. The analysis of all the groups of diseases shows that the laparoscopic procedures performed with 3D technology have a shorter mean operative time than comparable 2D procedures when we consider surgery that require complex tasks. The use of 3D laparoscopic technology is an extraordinary innovation in clinical practice, but the instrumentation is still not widespread. Precisely for this reason the studies in literature are few and mainly limited to the evaluation of the surgical skills to the simulator. This study aims to evaluate the actual benefits of the 3D laparoscopic system integrating it in clinical practice. The three-dimensional view allows advanced performance in particular conditions, such as small and deep spaces and promotes performing complex surgical laparoscopic procedures.

## INTRODUCTION

Since it's introduction, laparoscopic surgery represented a real revolution in clinical practice. In the last decades technological advances like high-definition (HD) cameras, dedicated instruments and articulating staplers, improved safety and feasibility of laparoscopic procedures. We saw a large diffusion of laparoscopic surgery with more difficult and complex operations. Nevertheless, laparoscopic surgery is more difficult to learn and requires different psychomotor skills than open surgery: the surgeons work in a three-dimensional space, but are guided by two-dimensional images. This limitation can be challenging, especially with regard to maneuvers requiring precision and dexterity [1]. The development of high definition cameras and articulating instruments did not eliminate the major limitations of two-dimensional (2D) laparoscopy: the lack of depth perception and the lose of spatial orientation with potential increasing of surgical strain, risk of errors and operative time. The spatial depth information loss in two-dimensional vision system was compensated by surgeon experience and by the ability of human brain to interpret spatial depth. Three-dimensional (3D) HD cameras was created as an alternative to conventional 2D laparoscopy [2]. Although 3D technology was introduced in the early 1990s its equipment is still not diffused on territorial hospitals because of initial observations of side effects when using 3D vision systems, poor image resolution and more expensive procedures [3]. In 1998 Hanna GB et al [4] showed that there were no advantages from use of 3D laparoscopic system. On the other side the development of robotic surgery in the recent years demonstrated the multiple advantages of a full immersion three-dimensional HD vision and, at the same time, the management problems derived from elevated costs and new learning curve of this kind of surgery. For these reasons, the use of a new generation three-dimensional (3D) HD laparoscopic system can be considered a favorable “hybrid” made by combining two different elements: improved quality of vision (3D from robotic surgery) and tactile feedback and proprioception (from laparoscopy). These two elements could seem to reduce learning curve with improved surgical precision. In literature there are still few clinical studies about use of 3D with different results [5]. Instead several experimental studies were performed using training boxes by comparing 3D with 2D imaging. These studies suggested a better surgical performance using 3D laparoscopy, such as shorter operative time and less number of errors [6, 7]. The studies carried out in the simulator by surgeons and medical students gave information on technical skills, but did not correspond to clinical reality that might present many different situations and subjective variables, with biased results. For example the duration of the procedures, the positioning of the trocars, the harmony and communication of the surgical team varied significantly. In this study we report our clinical experience with use of three-dimensional (3D) HD vision system for laparoscopic surgery.

## RESULTS

Table 1 shows the results of the two laparoscopic group. Considerating specific surgical procedures there is no significant difference in term of age and gender. The analysis of all the groups of diseases shows that the laparoscopic procedures performed with 3D technology have a shorter mean operative time than comparable 2D procedures. Really, statistical analysis demonstrates that there are no significant differences in terms of time for the procedures that do not require ligatures or intra-corporeal knotting such as adrenalectomy, nephrectomy and splenectomy. On the contrary, the differences become significant when we consider procedures that require complex tasks like anti-reflux surgery, treatment of achalasia, colo-rectal resection, gynecological surgery with in particular sacrocolpopexy. We observed no significant differences in terms of intra-operative bleeding and other complications. The rate of conversion is the same in the two groups.

**Table 1:** Type of surgery and patients data, results of mean operative time, blood loss, complications and conversion in 2D versus 3D laparoscopic surgery

Type of surgery	Patients data	2D	3D	p-value
Upper GI surgery (n= 46)	Mean age	42.3 (range 21 – 62)	0 (range 18 – 60)	<.05
achalasia (n= 30)	Gender (M:F)	19:27	20:26	
GERD (n= 16)	Mean operative time	115 min (80 – 145)	85 min (60 – 95)	
	Blood loss	NS	NS	
	Complications	2 esophageal perforation -	-	
	Conversion		-	
Adrenalectomy (n= 27)	Mean age	54.2 (range 38 – 74)	5.8 (range 42 – 72)	>.05
	Gender (M:F)	15:12	13:14	
	Mean operative time	120 min (100 – 240)	95 min (55 – 210)	
	Blood loss	splenic capsule lesion	NS	
		perirenal fat		
	Complications	-	-	
	Conversion	-	-	
Nephrectomy (n= 22 )	Mean age	59 (range 37 – 81)	63.2 (range 48 – 79)	>.05
	Gender (M:F)	12:10	13:9	
	Mean operative time	195 min (110 – 290)	170 min (95 – 310)	
	Blood loss	NS	NS	
	Complications	-	-	
	Conversion	-	-	
Splenectomy (n= 12 )	Mean age	42 (range 27 – 64)	45.5 (range 26– 58)	>.05
	Gender (M:F)	5:7	6:6	
	Mean operative time	135 min (95 – 240)	118 min (90 – 200)	
	Blood loss	NS	NS	
	Complications	-	-	
	Conversion	-	-	
Colo-rectal resections (n= 38 )	Mean age	61.5 (range 42 – 78)	58.3 (range 37 – 72)	<.05
	Gender (M:F)	23:15	22:16	
	Mean operative time	210 min (180 – 260)	160 min (120 – 210)	
	Blood loss	NS	NS	
	Complications	anastomotic leakage	-	
	Conversion	pelvic adhesions	-	
Gynecological surgery (n= 18 )	Mean age	2 (range 36 – 68)	53.6 (range 38 – 64)	<.05
	Mean operative time	125 min (80 – 155)	90.5 min (60 – 125)	
	Blood loss	NS	NS	
	Complications	-	bladder lesion	
	Conversion	-	-	

Upper GI surgery: we have not registered any complication in the treatment of reflux disease. In two patients undergoing esophageal myotomy for achalasia with 2D technology occurred esophageal perforation intraoperatively repaired with sutures and without post-operative complications. There were no esophageal lesion in the 3D group.

Adrenal surgery: in 2D group we recorded an intraoperative bleeding from the splenic capsule lesion and in another case a diffuse bleeding from the perirenal fat. Both of these conditions have been treated during surgery without resulting in a significant increase in estimated blood loss and without conversion to open procedure. There were no postoperative complications in any of the two groups.

Renal surgery: as above described, we considered only the cases of radical nephrectomy. In these patients there were no intraoperative complications or bleeding. The longest duration procedure was a 3D laparoscopic left nephrectomy with simultaneous para-aortic and inter-aortocaval extented lymphadenectomy for repetitive lesion (40/46 nodes isolated with metastatic lesions). The only significant bleeding (loss of 3 g/dl of Hb) has occurred in the case of a bilateral nephrectomy for polycystic kidney with multifocal neoplasm but this case is excluded from this study (Table 2).

**Table 2:** Parameters of evaluation of surgical team, residents and medical students during live surgery sessions

Surgical team parameters	Mean Score after 2D group	Mean Score after 3D group	p-value
Variables of surgical outcome^a^			
- Precision	3.2 (range 2–4)	4.6 (range 3-5)	<.05
- Definition of planes	3.0 (range 2–4)	4.8 (range 4–5)	<.05
- Depth perception	2.5 (range 1–4)	4.8 (range 4–5)	<.05
Variables of surgical strain^b^			
- Wrist and hand strain	3.1 (range 2–4)	2.8 (range 2–4)	NS
- Neck and back strain	1.8 (range 1-4)	1.6 (range 1–3)	NS
- Eye strain	2.8 (range 2–4)	3.0 (range 2–4)	NS
- Dizziness and/or headache	1	1.6 (1–2)	NS
**Residents and medical students**			
Variables of surgical outcome^a^			
- Precision	2.3 (range 2–4)	4.8 (range 4-5)	<.05
- Definition of planes	2.1 (range 2–3)	4.7 (range 4–5)	<.05
- Depth perception	1.8 (range 2–4)	4.8 (range 4–5)	<.05
Variables of surgical strain^b^			
- Wrist and hand strain	-	-	
- Neck and back strain	-	-	
- Eye strain	3.5 (range 2–4)	2.8 (range 2–4)	NS
- Dizziness and/or headache	1.4 (range 1–2)	1.8 (1–3)	<.05
What is your interest for the surgery?^c^	3.0 (range 1–5)	4.8 (range 2–5)	<.05

^a^Variables of surgical outcome: score 1–5; 5 = excellent.

^b^Variables of surgical strain: score 1–5; 1 = no strain.

^c^Question only for medical students.

Splenic surgery: No significant differences, none intra and postoperative complications.

Colo-rectal surgery: There were no intraoperative complications or significant bleeding in both group. In a case of rectal resection in 2D laparoscopic group we registered an anastomotic leakage in third POD treated conservatively with redo laparoscopic surgery, colic suture and packaging of lateral ileostomy. The subsequent postoperative course was regular in absence of other complication. At follow-up of two years the patient is free from disease. The only case of conversion was due to an extensive pelvic adherence syndrome in a patient with previous hysterectomy with extented laparotomic incision.

Gynecological surgery: The only intraoperative complication was a bladder lesion in 3D laparoscopic group for endometrial cancer. Despite this drawback and the need for additional intracorporeal sutures the operative time was lower than 2D laparoscopic group anyway. We have not recorded other intra and post-operative complications.

The surgical team reported better depth perception with the 3D system and a subjective reduction in the visual and muscle fatigue with 3D technology rather than with traditional laparoscopy. These benefits were evident mainly for the longer duration and complex procedures. The same perception was confirmed by medical students and residents who attended the live surgery session. Also from the questionnaires is indicated a greater interest in surgical procedures when these were made with 3D technology. Only in 16 cases, always in different students that use three-dimensional vision for the first time, we registered negative effects of 3D vision like headache and dizziness because of the time necessary for adaption to the stereo effect [8, 9] (Table 2)

## DISCUSSION

Although the 3D technology, in the last years, has greatly improved the laparoscopic vision, it is not still the standard for this type of surgical approach. The three-dimensional view allows advanced performance in particular conditions, such as small and deep spaces (mediastinum, retro-esophageal space, adrenal loggia, pelvic space) and promotes performing complex surgical laparoscopic procedures as sutures and intracorporeal knotting. In literature there are still few studies on the application of 3D laparoscopic technology and most of these are based on data obtained in simulator or box trainer [6, 7, 10, 11]. This study has the advantage of applying 3D technology to the most frequent abdominal surgical procedures. We considered the primary endpoints: mean operative time, intraoperative complications, estimated blood loss and post-operative complications. These are objective quantitative parameters of better surgical precision. The results allow us to state that the 3D technology improves surgical performance with statistically significant differences just in the most complex cases. We also wanted to emphasize the subjective perception of the individual members of the surgical team by submitting a questionnaire to assess their degree of visual and physical strain obtaining favorable results for the three-dimensional vision. In our University Hospital we administered a modified questionnaire for measuring surgical outcome and personal interest of a total of different 30 residents and medical students during live surgery sessions: 3D laparoscopic vision increased interest for surgical procedures. Only in 16 cases, always in different students that used three-dimensional vision for the first time, we registered negative effects of 3D vision like headache and dizziness. These results can be explained partially by analyzing literature data that indicate inability to stereoscopic vision as a variable from 1% to 30% in the general population and 9.7% among surgeons [12, 13]. In addition, the use of laparoscopic 3D technology requires some technical attention respect to traditional 2D laparoscopy: in particular the optics should be as stable as possible, with small movements carried out slowly, and we should minimize the ambient lightness of the operating room to enhance the vision and contrast of the 3D monitor [14, 15]. In this study we did not take into consideration the levels of communication between the medical and technical staff in the operating room. It is possible in fact that reducing mean operative times obtained with 3D technology are due not only to a strictly surgical factor, related to the improved depth perception, but also to greater interaction between the different component of the surgical team. So, the questionnaires administered to resident and medical students could be extended to anesthesiologists and nursing team. Our aim for the future will be to investigate this “indirect” aspect of the use of the 3D vision system. We have already started to collect data about physical and mental stress of the surgeon by periodically recording of the heart rate and blood pressure while performing the surgical procedure.

## MATERIALS AND METHODS

Between January 2013 and April 2017 a prospective cohort study was conducted at the Department of General and Emergency Surgery of the University Hospital Policlinico “P. Giaccone” of Palermo. We considered 163 patients who underwent to laparoscopic three-dimensional (3D) HD surgery for various indications. This 3D-group was compared to a retrospective-prospective control group of patients who underwent the same surgical procedures between January 2010 and April 2017 (2D-group). We used random selection for patients. All surgical procedures were performed by the same surgical team in order to attenuate the effect of learning curve and to obtain a standard technique.

The 2D imaging system consisted of KARL STORZ 2D/HD system equipped with a 30° direction of view and 10 mm diameter laparoscope and the 3D imaging system was KARL STORZ 3D Camera System (Karl Storz, Tuttlingen, Germany) equipped with a 0° and 30° direction of view and 10 mm diameter laparoscope and a 3D camera control unit for the transmission of 3D signals to a HD 3D monitor.

### Patients selection

**Figure 1:**
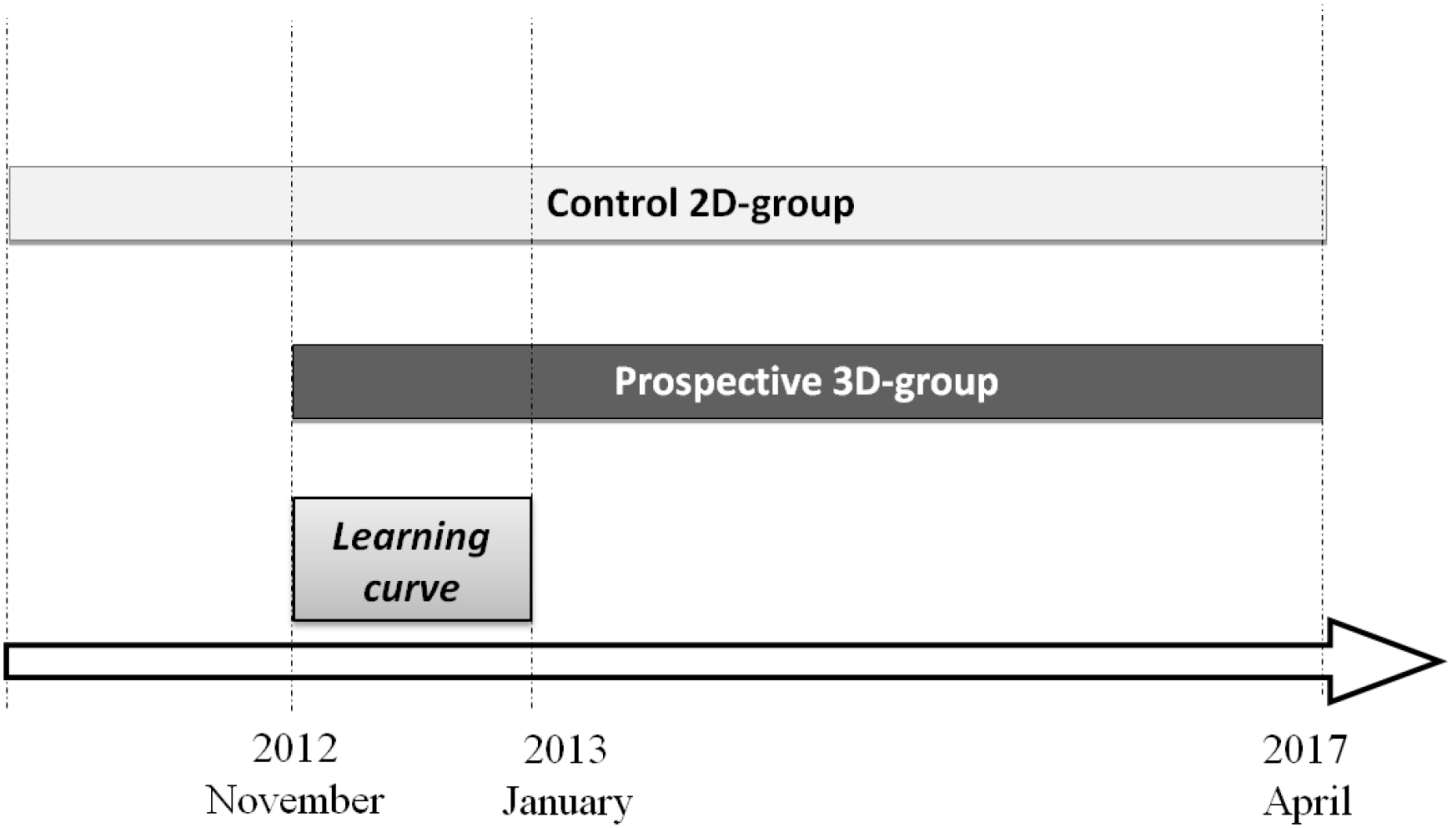
Study design We considered 163 patients underwent to laparoscopic three-dimensional (3D) HD surgery. This prospective 3D-group was compared with a retrospective-prospective control 2D-group of patients who underwent the same surgical procedures between January 2010 and April 2017. The patients were selected at ratio 1:1, for every patient in 3D-group, a patient with the same characteristics and pathology was selected from 2D-group. We used random selection for patients. In the learning curve period from November 2012 to January 2013 we performed intensive basic laparoscopy (cholecystectomies and appendectomies) in order to familiarize with 3D system.

The patients were selected at ratio 1:1, i.e., for every patient in 3D-group, a patient with the same characteristics and pathology was selected from 2D-group. This is a retrospective selection so we used the last procedures made with two-dimensional (2D) HD laparoscopic system. We used 3D system for the first time in November 2012 for laparoscopic cholecystectomy and appendectomy and, in the last four years, we carried out upper GI surgery, colo-rectal resections, adrenalectomy, radical and sparing renal surgery, splenectomies, gynecological surgery (Figure 1).

The variety of surgical operations could result in bias related to the heterogeneity of procedures therefore we identify three groups:

1. Basic Laparoscopy: 28 cholecystectomies and 13 appendectomies [16, 17]. They were made at the beginning of our experience and are part of the learning curve. They are not taken into account in this study. Before starting the study the surgeon and the entire surgical team familiarized with 3D system.
2. Advanced Laparoscopy that does not require specialized skills such as suturing, intra-corporeal knotting or other difficult surgical tasks. In this group we consider adrenalectomies, radical renal surgery and splenectomies.
3. Advanced Laparoscopy that needs specific laparoscopic skills such as suturing, intra-corporeal knotting and intestinal anastomosis. In this group we consider functional Upper GI surgery (esophageal achalasia and anti-reflux surgery), colo-rectal resections, gynecological surgery.

**Figure 2:**
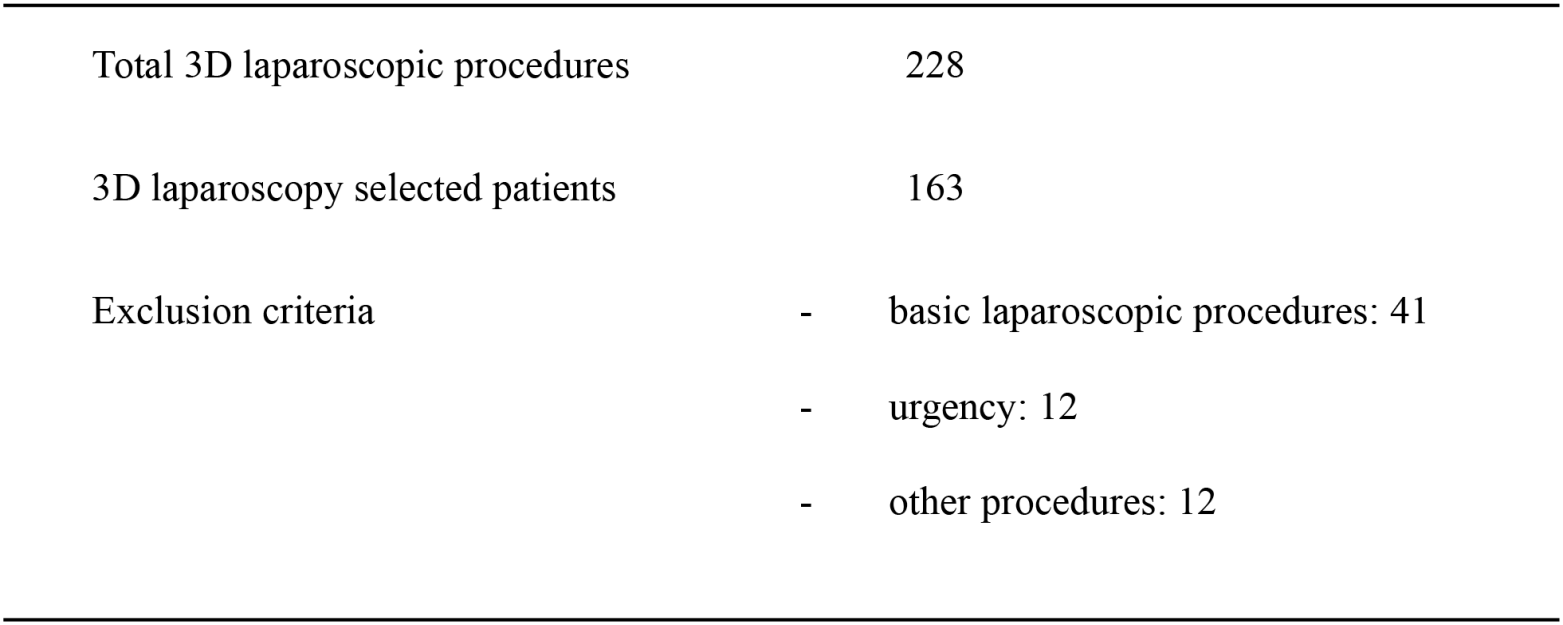
Patients treated with 3D laparoscopic surgery and exclusion criteria

In addition to the basic laparoscopy we also excluded from 3D-group no standardized procedures such as nephron-sparing surgery (6 cases) and bilateral nephrectomy con polycystic kidney (2 cases), a case of splenectomy with en bloc resection of gastric fundus and diaphragmatic surface, a case of laparoscopic resection of epiphrenic esophageal diverticulum, a case of anterior resection of the rectum with trans-anal TME (TaTME), one laparoscopic posterior mesh rectopexy for prolapse, and urgent surgical procedures like three gastric perforation, five stenotic colonic neoplasms, two perforated diverticulitis, one splenectomy for trauma and a right nephrectomy for large bleeding angiomyolipoma [18, 19] (Figure 2). In the retrospective 2D-group we use the same exclusion criteria.

### Surgical technique

In both two groups we used the same standard surgical technique. In all cases we used Veress needle to induce pneumoperitoneum.

Functional Upper GI surgery: Patients were placed on the operating table in the reverse Trendelemburg position with legs apart and slightly bent (the classic French position). Five trocars were positioned for this kind of surgery. In treatment of achalasia the esophagus gastric junction access was created from left to right side with sectioning of the phrenoesophageal membrane. We prepared the distal esophagus on the anterior wall. We did not prepare the posterior wall. The myotomy began on the esophagus and proceeded towards above 4-6 cm, before passing to the gastric side for another 2 cm. A 180° Dor fundoplication was performed for the dual purpose of controlling gastroesophageal reflux and protecting the esophageal submucosa [20, 21]. In the treatment of reflux disease left and right crura were exposed and a retroesophageal window was created. After correct and complete diaphragmatic pillars exposition we given nonassorbable suture to primary closure of the esophageal hiatus. In our experience we used also a Gore Bio-A Tissue reinforcement adsorbable mesh with a “U” shape to reinforce hiatoplasty [22]. In the end a Nissen fundoplication was realized with a wrap of 2 cm long.

Laparoscopic adrenalectomies: Were performed by a transperitoneal flank approach in the lateral decubitus position with an inclination of 50-60° relative to the operating table which is broken to extend the space between the last rib and the iliac crest. For right adrenalectomy we used four trocars in the right subcostal region. The right lobe of the liver is mobilized by division of triangular ligament. Tissue dissection along the lateral border of the inferior vena cava allowed the identification of the short adrenal vein which was clipped and divided. For left-side resection we positioned three trocars in the left subcostal region. We divided splenocolic and splenophrenic ligament. The spleen and pancreatic tail were rotated medially. The peritoneal dissection was performed until the left renal vein is reached. We used also diaphragmatic vein like landmarks for adrenal vein which was clipped and divided [23–30].

Laparoscopic nephrectomy: We used the same approach of adrenal surgery. In right nephrectomy tissue dissection along the lateral border of the inferior vena cava allowed the identification of the renal vein and posterior renal artery. In left renal surgery, after spleno-pancreatic mobilization, we found renal vein from gonadic vessel. The artery is always posterior in our experience [31].

Laparoscopic splenectomy: Was conducted using lateral approach and four trocars in subcostal region. Then mobilization of the splenic flexure of the colon we opened gastrosplenic ligament to indentify and dissect splenic artery and splenic vein. The short gastric vessels are divided with harmonic scalpel. The next step was progressive mobilization of the spleen by division of the posterior and superolateral peritoneal attachments.

Colo-rectal resections: The patient was placed on the operating table in the Trendelemburg position with leg apart. We performed right hemicolectomies with intra-corporeal anastomosis, left hemicolectomies and rectal resections. In right hemicolectomy we used a 10 mm trocar for laparoscope in left peri-umbilical region and other three trocars rispectively in left upper and lower quadrant (5 mm) and in sovra-pubic region (12 mm) for endo-stapler. We started with the identification of the ileum-colic vessels. Later we made the preparation of the last ileal loop and the colo-epiploic detachment. We performed a side to side intracorporeal anastomosis with endo-stapler and a continous riassorbable suture with intra-corporeal knotting. In left hemicolectomies and rectal cancer resections we used optical trocar in the right peri-umbilical region and other three trocars in the right iliac fossa (12 mm for endo-stapler), in the right upper quadrant and left flank (5 mm) The surgical technique used involved the initial colo-epiploic detachment. Then we proceeded with isolation of inferior mesenteric vein by disconnecting the plan between Toldt and Geroata fascia. So we clipped and divided inferior mesenteric artery. For anterior resection of the rectum approach to mesorectum with running TME began on the back plane and then to the sides and front. We have always realized an intra-corporeal end-to-end colorectal anastomosis.

Gynecological surgery: We placed the patients in dorsal lithotomy and steep Trendelenburg position. Clermont-Ferrand uterine manipulator (Karl Storz, Tuttlingen, Germany) was routinely used for adequate pelvic exposure with a 10-mm trocar positioned at the umbilicus for the camera and other three trocars at each lower abdominal quadrant and in sovrapubic area. Laparoscopic hystero-salpingooophorectomy with bilateral pelvic lymph node dissection was performed in selected patients with stage I endometrial cancer. Lymph nodes were removed transvaginally altoghether after the completion of hysterectomy without spillage into the trocars or in the peritoneal cavity [32]. In patient with symptomatic apical prolapse we performed laparoscopic sacrocolpopexy. The process began with the identification of the promontory of the sacrum with opening of posterior peritoneum until rectum region. We dissected the rectovaginal space to facilitate posterior access to the levator ani. Then we placed a non-absorbable Y polypropylene mesh, with prolene 2-0. The two branches of the Y mesh were attached to the anterior and posterior vagina walls with prolene 2-0. Then the long branch of the mesh was fixed to the sacral promontory with one or two non-absorbable sutures. Laparoscopic sacrocolpopexy is an advaced technical procedure that needs specific laparoscopic skills such as suturing, intra-corporeal knotting and prosthesis position [33].

### Evaluation of surgical outcome

In this study we analyzed the use of three-dimensional (3D) HD vision system in the Advanced Laparoscopic surgery for some reasons:

- particular deep location or small spaces such us renal and adrenal loggia, pelvic space and retroesophageal window or anterior and posterior mediastinum with theoretical maximum advantage of a 3D system;
- surgical technique that request specific advanced laparoscopic skills such as suturing, intra-corporeal knotting and intestinal anastomosis.

We considered like primary end-points operative time, intraoperative estimated blood loss and other intra-operative complications, conversion rate [34]. These parameters are direct objective signs of surgical precision. In this case differences between the two groups for variable were determined by x^2^ exact test and Student *t* test. Statistical significance was considered P <0.05. we did not evaluate hemodynamics and psycomental stress parameters of the surgeon [35]. We registered postoperative complication. None of the complications could be assigned specifically to 3D visualization. We instead considered also other subjective variables with use of a questionnaire for surgical team with the scope of evaluate quality of depth perception and surgical strain. The variables analyzed for surgical outcome were: precision, definition of planes, stereopsis and depth perception. The variables for surgical strain were: wrist and hand strain, back strain, neck strain and eye strain [36]. In our University Hospital we also administered a modified questionnaire for measuring surgical outcome and personal interest of a total of different 30 residents and medical students during live surgery sessions [37].

## CONCLUSIONS

The use of 3D laparoscopic technology is today an extraordinary innovation in clinical practice, especially during the execution of the most complex procedures. Despite these assumptions, the instrumentation is still not widespread, mostly used in high volume centers. Precisely for this reason the studies in literature are few and mainly limited to the evaluation of the surgical skills to the simulator. This study, along with a few others, aims to evaluate the actual benefits of the 3D laparoscopic system not limiting it to the execution of single surgical tasks, but integrating it in clinical practice. For this reason we analyzed several surgical procedures that required different knowledge and manual skills and we involved different medical “extraneous” figures (medical students, postgraduate resident, endocrinologists, radiologists, gastroenterologists). The analysis performed shows an objective and subjective benefit of using this technology, but further studies are needed to validate the results.
